# Anti-adipogenic effects of the traditional herbal formula Dohongsamul-tang in 3T3-L1 adipocytes

**DOI:** 10.1186/s12906-017-2038-z

**Published:** 2017-12-19

**Authors:** Eun Hyoung Shim, Hoyoung Lee, Myeong Soo Lee, Sooseong You

**Affiliations:** 1 KM Fundamental Research Division, Korea Institute of Oriental Medicine 483 Expo-ro, Yuseong-gu, Daejeon, 305-811 Republic of Korea; 2 Clinical Research Division, Korea Institute of Oriental Medicine 483 Expo-ro, Yuseong-gu, Daejeon, 305-811 Republic of Korea

**Keywords:** Dohongsamul-tang, Blood stasis syndrome, Anti-adipogenesis, Metabolic syndrome, Herbal medicine

## Abstract

**Background:**

Blood stasis syndrome (BSS) is a general pattern identification and refers to pathological stagnation of blood circulation, dysfunction of endothelial cells or metabolic disorder in traditional Korean medicine (TKM). Dohongsamul-Tang (DHSMT) is a well-known traditional herbal formula which used for treatment and prevention of BSS by promoting blood circulation in TKM.

**Methods:**

Cytotoxicity of DHSMT was examined by cell counting kit-8 (CCK-8). We also investigated the anti-adipogenesis effect of DHSMT by using Oil Red O staining, intracellular triglyceride assay leptin ELISA and western blot analysis in 3T3-L1 adipocytes. In addition, the accumulation of adiponectin, resistin and plasminogen activator inhibitor-1 (PAI-1) were measured by magnetic bead panel kit.

**Results:**

Oil Red O staining showed that DHSMT markedly reduced fat accumulation without affecting cell cytotoxicity. DHSMT also significantly decreased accumulation of triglyceride and adipokines such as leptin, adiponectin, resistin and PAI-1 compared with fully differentiated adipocytes. Furthermore, our results found that DHSMT significantly suppressed the adipocyte differentiation by downregulating adipogenic-specific transcriptional factors such as peroxisome proliferator-activated receptor gamma (PPARγ), CCAAT/enhancer binding proteins alpha (C/EBPα) and fatty acid binding protein 4 (FABP4) in adipocytes.

**Conclusions:**

Taken together, our findings provide that DHSMT has potential for treatment and prevention of obesity or MS related to BSS.

## Background

Metabolic syndrome (MS) is accompanied with risk factors such as disorder of glucose metabolism, obesity, dyslipidemia, hyperglycemia, hypertension, diabetes mellitus or insulin resistance with two or more conditions occurring concurrently [1–3]. Obesity results from an imbalance between caloric intake and expenditure and is characterized by an increased risk of MS, including hypertension, cardiovascular disease, and type 2 diabetes [4]. Blood stasis syndrome (BSS) is an important pathological concept in traditional Korean medicine (TKM) that was first recorded in *Huangdi’s Inner Classic* [5]. In recent years, several studies have reported that BSS is related to MS and its risk factors such as obesity, atherosclerosis, hypertension and diabetes mellitus [6–8]. Several herbal formulas such as Dohongsamul-Tang (DHSMT), Doinseunggi-Tang, Sobokchukeo-Tang, Hyeolbuchukeo-Tang have been widely used for treating BSS by circulating blood flow in TKM. Notably, DHSMT, which was first recorded in *The Golden Mirror of Medicine*, is a traditional herbal formula containing a*ngelis gigantis radix, persicae semen, rehmanniae radix, cnidii rhizome,* and *carthami flos*. And, DHSMT promotes blood circulation according to TKM and has been used to treat BSS, dysmenorrhea, contusion, abnormally colored menses, and menostasis [9]. To date, several studies have reported the effects of DHSMT, which include an anti-trombotic effect [10], an anti-inflammatory effect [11, 12], and relief from endometriosis [13]. However, the mechanism of action of DHSMT is still unclear. There are few studies available that explain the mechanism of action of DHSMT. Therefore, we evaluated its potential effects on anti-adipogenesis, regulation of transcription factors related to adipogenesis of 3T3-L1 adipocytes.

## Methods

### Materials

The mouse fibroblast cell line, 3T3-L1 cells were obtained from the American Type Culture Collection (ATCC, Manassas, VA, USA) and Dulbeco’s modified eagle’s medium (DMEM), fetal bovine serum (FBS), newborn calf serum (NBCS), penicillin-streptomycin (P&S) and Dulbeco’s phosphate-buffered saline (DPBS) were obtained from Gibco BRL. (NY, USA). Dimethyl sulfoxide (DMSO), formaldehyde, dexamethasone (DEX), 3-isobutyl-1-methylisobutylxanthine (IBMX), triton X-100 and Oil Red O staining powder were purchased from Sigma-Aldrich (St. Louis, MO, USA) and the cell counting kit-8 (CCK-8) was purchased from Dojindo Laboratories (Kumamoto, Japan). Trigliceride (TG) kit was obtained from Bioassay Systems (CA, USA) and leptin ELISA kit was purchased from R&D System Inc. (MI, USA). Milliplex^®^ MAP mouse adipocyte magnetic bead panel kit was obtained from Millipore Co. (MA, USA). Antibodies against proliferator-activated receptor gamma (PPARγ) and fatty acid binding protein 4 (FABP4) were purchased from Cell Signaling Technology Inc. (Beverly, MA, USA), CCAAT/enhancer binding proteins alpha (C/EBPα) and β-actin were purchased from Santa Cruz Biotechnology Inc. (CA, USA). The anti-mouse or anti-rabbit secondary antibody attached to horseradish-peroxidase-conjugate were obtained from Bio-Rad Laboratories Inc. (PA, USA). All other reagents from commercial sources were condition of analytical grade.

### Preparation of herbal extracts

DHSMT composed of each five different types of herbs including a*ngelis gigantis radix, persicae semen, rehmanniae radix, cnidii rhizome, carthami flos* (Table 1). Each herbs were obtained from from a traditional herb market, Omniherb (Daegu, Korea) in 2012 and medicinal herbs crushed by grinder were extracted by heating in distilled water for 3 h at 100 °C using reflux extraction (COSMOS-660, Kyungseo Machine Co. Incheon, Korea). After then, DHSMT was concentrated by using vacuum evaporator (EYELA N-12 EYEKA CA-1112, Tokyo, Japan) and was freeze-dried (PVTFD-100, ilShinBioBase, Gyeonggi-do, Korea). The herbal components were identified by Dr. Jun-Kyung Lee of Hyemin Dispensary of Oriental Medicine (Jeonju, Korea). The voucher specimen (BS-2) and each herbal components were stored at the Korea Medicine Fundamental Research Division, Korea Institute of Oriental Medicine (Daejeon, Korea).

**Table 1:** Prescription of Dohongsamul-Tang (DHSMT)

Crude drug		Original region	Dosage (g)
Herbal name	Scientific name		
Angelis gigantis radix	Angelica gigas Nakai	Korea	16.00
Persicae semen	*Prunus persica* Batsch	China	16.00
Rehmanniae radix	Rehmannia glutinosa Libosch	Korea	12.00
Cnidii rhizome	Cnidium officinale Makino	Korea	8.00
Carthami flos	*Carthamus tinctorius* Linne	Korea	8.00
Total (g)			60.00
Yield (%)			14.81

### High performance liquid chromatography (HPLC) analysis

The lyophilized extract (10 mg) was dissolved in 70% methanol (5 ml) and then filtered through a 0.2 μm membrane filter (Woongki Science Co., Ltd., Seoul, Korea) before being injected into HPLC for component analysis. The purity of the ten standard compounds was ≥98.0% using HPLC analysis. The HPLC grade solvents, methanol, acetonitrile and water were obtained from J.T.Baker (Phillipsburg, NJ, USA). Trifluoroacetic acid (analytical reagent grade) and the standards were procured from Sigma-Aldrich (Merck Millipore, Darmstadt, Germany). The HPLC system consisted of a Waters Alliance 2695 system coupled with a 2998 photodiode array detector (Waters Corporation, Mitford, MA, USA). Data processing was performed with Empower software, version 3 (Waters Corporation, Milford, MA, USA). The 5 components in DHSMT were separated using a Luna 5 μm C18 100A column (4.6 × 250 mm, 5 μm particle size, no. 00G-4252-E0; Phenomenex, Inc., Torrance, CA, USA). The monitoring was performed at 330 nm and 400 nm for three compounds (nodakenin, ferulic acid and sophoricoside) and two compounds (safflomin A and quercetin), respectively. The mobile phases consisted of water with 0.1% (*v*/v) trifluoroacetic acid (solvent A) and acetonitrile (solvent B) at a flow rate of 1.0 ml/min. The gradient conditions changed as presented in Table 2. The injection volume was 10 μl.

**Table 2:** Composition of mobile phase for chromatographic separation

Time (min)	Solvent^a^ (%)	Solvent^b^ (%)
0	95	5
30	40	60
40	0	100
45	0	100
50	95	5
60	95	5

^a^0.1% (*v*/v) trifluoroacetic acid in water

^b^acetonitrile

### Cell culture and differentiation

The mouse fibroblast cell line, 3T3-L1 cells were cultured in DMEM containing 10% NBCS and 1% P&S at 37 °C in a humidified atmosphere with 5% CO_2_. For cell differentiation, 3T3-L1 cells were seeded in growth media to full confluence. After confluence, cells were replaced to differentiation medium: DMEM containing 10% FBS, 1% P&S and a mixture of 0.5 mM IBMX, 1 uM dexamethasone, 1 μg/ml insulin (MDI), and treated with various concentration of DHSMT and 10 μM of SB203580 used as a positive control for 48 h (from day 0 to day 2). At this time, the cells were changed with DMEM containing 1 μg/ml insulin but no IBMX or DEX and treated with various concentration of DHSMT and SB203580 for following 72 h (from day 2 to day 5). After then, the medium was replaced and treated with DHSMT and SB203580 for the following 48 h (from day 5 to day 7).

### Cell cytotoxicity

The cell viability was examined by CCK-8. 3T3-L1 cells were seeded in 96-well plates and treated with various concentrations (0, 10, 20, 50, 100, 200, 500 and 1000 μg/mL) of DHSMT for 48 h. The absorbance was measured at 450 nm using a Benchmark Plus microplate reader (Bio-Rad Laboratories Inc., CA, USA) and the percentages of cell viability were calculated.

### Oil red O staining and fat droplets quantification

After cell differentiation, cells were stained with Oil Red O solution containing 0.3% Oil Red O in 60% isopropanol to measure fat droplets in adipocytes. Differentiated cells were washed with DPBS and fixed with 10% formalin for 1 h and stained with Oil Red O solution for 30 min at room temperature. After then, cells were washed three times with distilled water and visualized by microscopy (Olympus, Tokyo, Japan). To determine lipid accumulation, stained lipid droplets were dissolved in 100% DMSO and quantified by measuring the optical absorbance at 530 nm using a Benchmark Plus microplate reader.

### TG, leptin and adipokines production on adipogenesis

TG, leptin and adipokines production were measured after finishing cell differentiation in the presence or absence of DHSMT. Cell lysates were used to determine the TG (Bioassay Systems, CA, USA) quantification and supernatant was analyzed by according manufacturer’s protocols for leptin immunoassay (R&D System Inc., MI, USA). Adipokines production such as adiponectin, resistin and plasminogen activator inhibitor-1 (PAI-1) was measured using a Milliplex® MAP mouse adipocyte magnetic bead panel kit (MADCYMAG-72 K, Millipore Co. USA). Briefly, cultured supernatant was collected from the differentiated adipocytes which were treated in the presence or absence of DHSMT. Signal values were detected on a Bioplex® 200 system and Bioplex pro II wash station (Luminex, xMAP® Technology, Texas, USA) by according manufacturer’s protocols. Each samples were analyzed by the Bio-Plex® 200 system and adipokine concentrations were calculated by using a standard curve.

### Western blot analysis

Differentiated cells were washed twice with cold DPBS, harvested using a cell scraper and lysed with RIPA cell lysis buffer containing 0.5 M Tris-HCl, pH 7.4, 1.5 M NaCl, 2.5% deoxylcholic acid, 10% NP-40, 10 mM EDTA. And then, cell lysates were centrifuged at 13,000 rpm for 15 min at 4 °C. Protein concentration was measured with using the BCA protein assay kit (Thermo Fisher Scientific Inc., Rockford, IL, USA). Each proteins present in cell lysates were separated on 4–20% Criterion™ TGX™ precast Gel (Bio-Rad Laboratories Inc., PA, USA) electrophoresis and transferred onto the polyvinylidene fluoride membrane (PVDF, Amersham Pharmacia Biotech, Little Chalfont, UK). The membrane was then blocked for 1 h at room temperature with 5% skim milk and incubated with 1:1000 dilutions of each different primary antibodies for overnight at 4 °C. After then, membrane was incubated with horseradish-peroxidase-conjugate anti-mouse or anti-rabbit secondary antibodies (1:3000 dilutions) for 1 h at room temperature, and immunoreactive proteins were detected with the ECL kit (Thermo scientific, Rockford, UK). Bands were visualized by using CemiDoc™ XRS+ image analyzer (Bio-Rad Laboratories Inc., PA, USA).

### Statistical analysis

All data results are indicated as means ± SEM and all determination were repeated triplicate. The one-way analysis of variance (ANOVA) by Bonferroni multiple comparison method (SYSTAT 13.0 SPSS Inc. U.S.A) was used to evaluate the difference among multiple group. The *p*-value <0.05 was considered statistically significant.

## Results

### Evaluation of the cytotoxic effects of DHSMT in 3T3-L1 cells

**Fig. 1:**
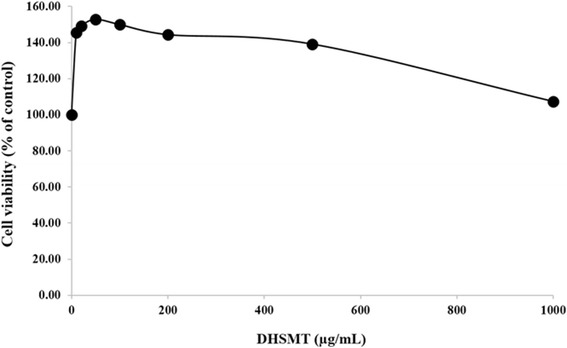
Cytotoxic effects of DHSMT in 3T3-L1 cells. 3T3-L1 cells were treated with various concentrations of DHSMT (0, 10, 20, 50, 100, 200, 500 and 1000 μg/mL) for 48 h. Cell cytotoxicity was measured by using CCK-8 kit and the absorbance was measured at 450 nm. The data are mean values of three experiments ± SEM

The yield of DHSMT was 14.81% (*w*/w) after freeze-drying. We evaluated the possible cytotoxicity of DHSMT in 3T3-L1 cells by using the CCK-8 assay. The cells were treated with various concentrations of DHSMT (0, 10, 20, 50, 100, 200, 500 or 1000 μg/mL). DHSMT has no significant cytotoxic effect in 3T3-L1 cells (Fig. 1). Therefore, further studies used a range of non-cytotoxic concentrations (62.5, 125, 250 and 500 μg/mL).

### HPLC analysis of DHSMT

**Fig. 2:**
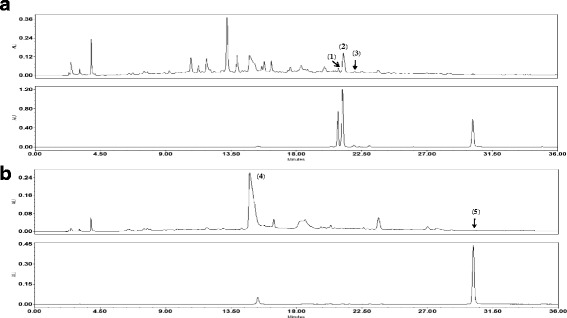
HPLC chromatogram standard mixture and DHSMT. **a** 330 nm and **b** 400 nm. (1) nodakenin, (2) ferulic acid, (3) sophoricoside, (4) safflomin A, and (5) quercetin

The results were obtained using mobile phases consisting of 1.0% (*v*/v) trifluoroacetic acid (solvent A) and acetonitrile with 1.0% (v/v) trifluoroacetic acid (solvent B). Quantitation was achieved using photodiode array detection in the region 200–400 nm based on the retention times and UV spectra compared with the standards. The UV absorbance was recorded at 330 nm for three compounds and 400 nm for two compounds. The retention times of compounds were 21.09 (nodakenin), 21.34 (ferulic acid), 21.84 (sophoricoside), 15.31 (safflomic A) and 30.21(quercetin) min, respectively (Fig. 2).

### Effect of DHSMT on lipid and leptin accumulation in adipocytes

**Fig. 3:**
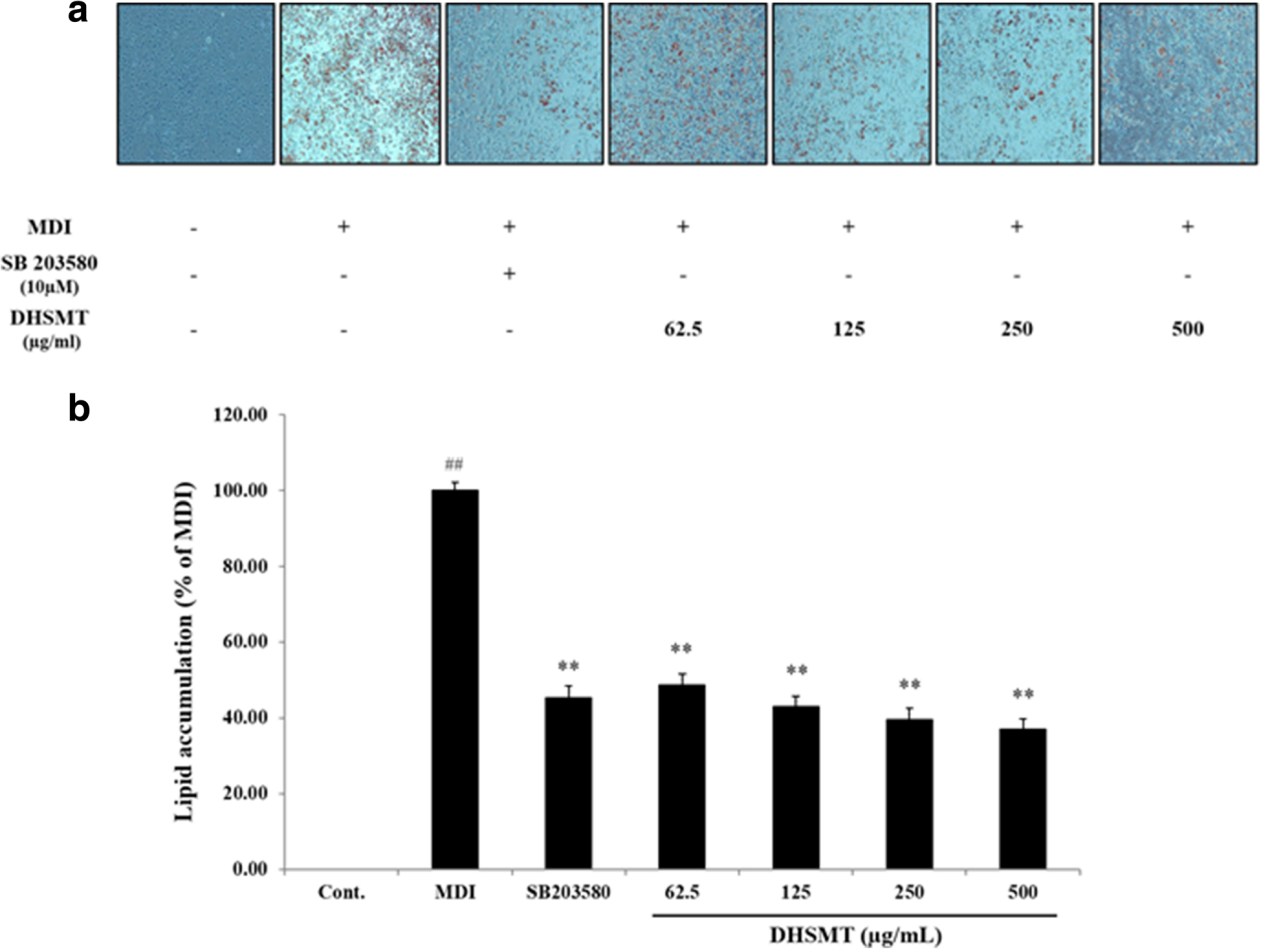
Inhibitory effect of DHSMT on the lipid accumulation in 3T3-L1 adipocytes during adipogenesis. 3T3-L1 cells were differentiated into adipocytes by incubation with a mixture of 3-isobutyl-1-methylisobutylxanthine, dexamethasone and insulin (MDI) and exposed to various concentrations of DHSMT (0, 62.5, 125, 250 and 500 μg/ml) and SB203580 for 7 days. **a** Lipid accumulation in cells were evaluated by Oil red O staining and visualized using microscope at ×100 of magnification. **b** Stained lipid droplet was dissolved in DMSO and quantified by reading the absorbance at 530 nm. The data are mean values of three experiments ± SEM; ## < 0.01 compared with control, *P < 0.05; **P < 0.01 compared with the MDI

**Fig. 4:**
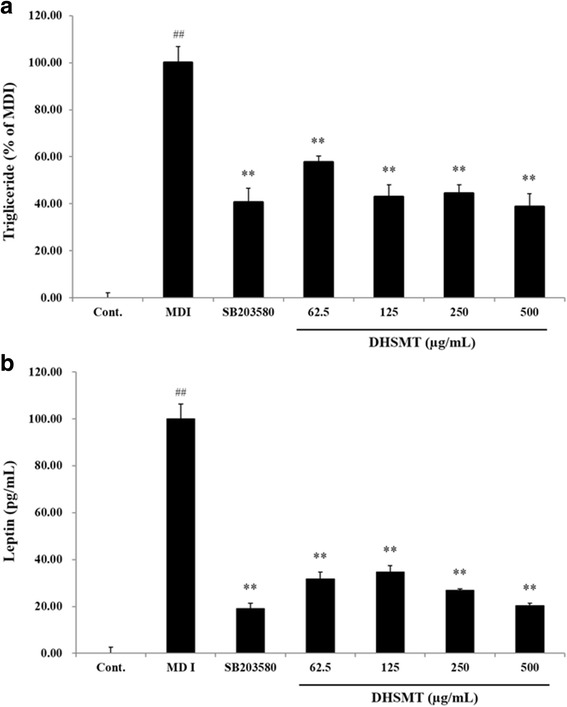
Inhibitory effect of DHSMT on triglyceride (TG) and leptin accumulation in 3T3-L1 adipocytes. 3T3-L1 preadipocytes were differentiated into adipocytes by using a mixture of 3-isobutyl-1-methylisobutylxanthine, dexamethasone and insulin (MDI) with various concentrations of DHSMT (0, 62.5, 125, 250 and 500 μg/ml) and SB203580 for 7 days. **a** TG content was measured enzymatically by using TG assay kit (Bioassay Systems, CA, USA). **b** Leptin production was measured at 450 nm by using a mouse leptin immunoassay kit (R&D Systems). The data are mean values of three experiments ± SEM; ## < 0.01 compared with control, *P < 0.05; **P < 0.01 compared with the MDI

To evaluate intracellular lipid accumulation in 3T3-L1 adipocytes, we performed Oil Red O staining. As shown in Fig. 3a, fat droplets significantly increased after differentiation for 7 days. However, fat droplets in cells treated by DHSMT and SB203580 decreased compared with the fully differentiated adipocytes. SB203580 is a selective inhibitor of p38 MAPK. This compound suppresses early adipogenesis by inhibiting the activation of p38 MAPK involved in adipocyte differentiation [14, 15]. In the present study, we used SB203580 as a positive control to confirm the efficacy on adipocyte differentiation of DHSMT. To quantify the lipid accumulation, fat droplets were dissolved by DMSO. Similar to Oil Red O staining, DHSMT significantly suppressed the lipid accumulation in a dose-dependently manner (*p* < 0.01 vs. MDI; Fig. 3b). Moreover, these results are consistent with the results that the TG content of cells in the presence or absence of DHSMT decreased 42.39 ± 2.78% (62.5 μg/mL), 57.09 ± 5.22% (125 μg/mL), 55.66 ± 3.67% (250 μg/mL) and 61.25 ± 5.63% (500 μg/mL) (*p* < 0.01 vs. MDI; Fig. 4a). DHSMT also significantly reduced leptin production (68.26 ± 2.99% (62.5 μg/mL), 65.27 ± 2.74% (125 μg/mL), 73.05 ± 0.60% (250 μg/mL) and 79.64 ± 1.04% (500 μg/mL)) compared with fully differentiated cells (*p* < 0.01 vs. MDI; Fig. 4b). The positive control, SB203580, dramatically inhibited adipogenesis in 3T3-L1 cells (*p* < 0.01 vs. MDI).

### Effect of DHSMT on the formation of adipokines in adipocytes

**Fig. 5:**
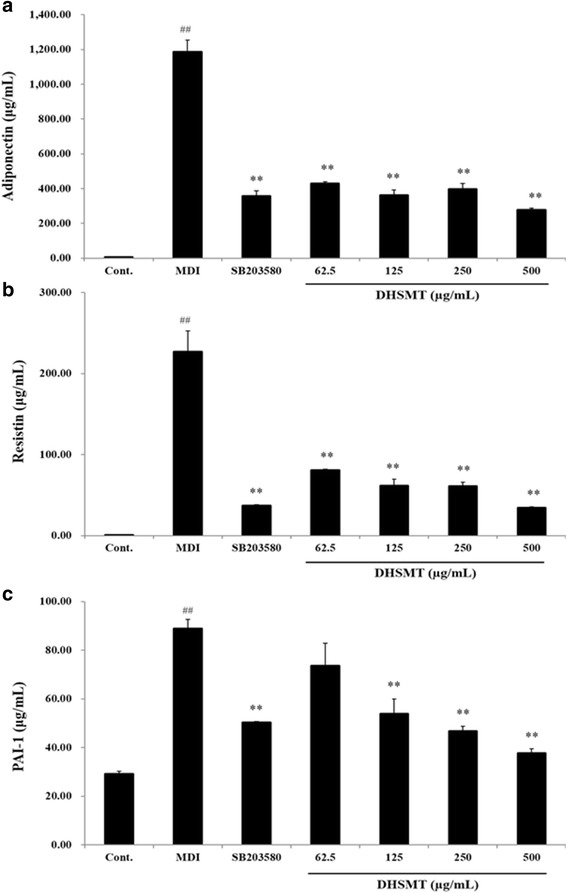
Inhibitory effect of DHSMT on adipokines accumulation in 3T3-L1 adipocytes. 3T3-L1 preadipocytes were differentiated into adipocytes by various concentrations of DHSMT (0, 62.5, 125, 250 and 500 μg/ml) and SB203580 with a mixture of 3-isobutyl-1-methylisobutylxanthine, dexamethasone and insulin (MDI) for 7 days. Adiponectin (**a**), resistin (**b**) and PAI-1(**c**) production were measured by a Milliplex^®^ MAP mouse adipocyte magnetic bead panel kit (Millipore Co. USA). The data are mean values of three experiments ± SEM; ## < 0.01 compared with control, *P < 0.05; **P < 0.01 compared with the MDI

To determine the effect of DHSMT on the formation of adipokines in fully differentiated adipocytes, we performed the multiplex assay for adiponectin, resistin and PAI-1 by using the supernatant. As shown in Fig. 5, DHSMT significantly decreased the levels of adiponectin. The concentrations of adiponectin were 430.25 ± 9.60 μg/mL, 360.75 ± 32.96 μg/mL, 396.42 ± 32.61 μg/mL and 277.99 ± 9.77 μg/mL at DHSMT concentrations of 62.5, 125, 250 and 500 μg/mL, respectively (*p* < 0.01 vs. MDI; Fig. 5a). The concentrations of resistin were 80.44 ± 1.25 μg/mL, 61.61 ± 8.05 μg/mL, 61.04 ± 5.13 μg/mL and 34.36 ± 1.26 μg/mL at DHSMT concentrations of 62.5, 125, 250 and 500 μg/mL, respectively (*p* < 0.01 vs. MDI; Fig. 5b). The concentrations of PAI-1 were 73.62 ± 9.25 μg/mL, 53.82 ± 6.13 μg/mL, 46.77 ± 1.95 μg/mL and 37.58 ± 1.95 μg/mL at DHSMT concentrations of 62.5, 125, 250 and 500 μg/mL, respectively (Fig. 5c). The positive control SB203580, also suppressed the release of adiponectin, resistin and PAI-1 (*p* < 0.01 vs. MDI). We also confirmed that DHSMT at 500 μg/mL decreased the levels of adiponectin, resistin and PAI-1 more than SB203580.

### Effect of DHSMT on the expression of adipocyte-specific transcription factors during adipogenesis

**Fig. 6:**
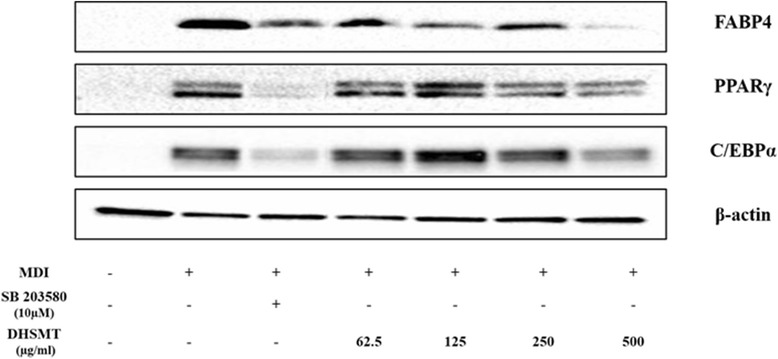
Effects of DHSMT on protein expressions of adipogenic-transcription factor during adipogenesis. 3T3-L1 adipocytes were exposed to various concentrations of DHSMT and SB203580 during the differentiation period. Cell lysates was isolated and protein expressions of PPARγ, C/EBPα and FABP4 were determined by western blot analysis. *ß*-actin was used as a control

Adipogenesis is accompanied by the activation of various adipogenic transcription factors and adipocyte-specific genes. To elucidate the mechanism of inhibition of adipogenesis by DHSMT, differentiated cells were treated with various concentrations of DHSMT. The protein expression of adipocyte-specific transcriptional factors were analyzed by western blotting. As shown in Fig. 6, DHSMT significantly suppressed adipocyte-specific markers, such as PPARγ, C/EBPα and FABP4. Notably, the decrease in PPARγ and C/EBPα expression was dose-dependent. We also found that 500 μg/mL DHSMT dramatically reduced the protein expression of FABP4.

## Discussion

BSS, called eohyul in Korea and yuxue in China, refers to the blood circulation is stagnant or blood flow is not smooth. The classical concepts of BSS were recorded as “blood and vessel stasis”, “retained blood” and “vascular obstruction” [5, 16]. BSS may also be related to the following conditions: disturbance in blood circulation and microcirculation, dysfunction of endothelial cells, metabolic disorder, and inflammation [17]. In recent decades, there have been many clinical studies correlating BSS and MS, including atherosclerosis, obesity, hypertension, coronary artery lesions, cardiac function, lipidemia, and diabetes mellitus [18–20].

Obesity, a metabolic disorder, significantly increases the risk of MS with its associated risk factors, such as atherosclerotic cardiovascular disease, diabetes, dyslipidemia, hypertension and other health problems [3, 21, 22].

In the present study, we evaluated the anti-adipogenic efficacy of a water extract of the traditional herbal formula DHSMT in MDI-induced 3T3-L1 adipocytes.

HPLC analysis is conveniently and widely methods to identify constituents of herbal plants in TKM [23]. We analyzed five main components of DHSMT using HPLC. The five main components were as follow: nodakenin from Angelis gigantis radix, ferulic acid from Rehmanniae radix, sophoricoside and safflomin A from Carthami flos, and quercetin from Cnidii rhizome. The established HPLC analysis method will be helpful for improving the quality control of DHSMT.

Oil Red O staining and the TG assay were used to determine whether DHSMT could alter TG production during adipogenesis. Our data revealed that lipid droplets containing TG were markedly increased in adipocytes. But, DHSMT significantly decreased the morphological differentiation of preadipocytes and TG accumulation in adipocytes without cytotoxicity.

Adipokines such as leptin, adiponectin, resistin and PAI-1 are physiologically active cytokines secreted from adipocytes that play an important role in the pathogenesis of MS through inflammation associated with obesity, atherosclerosis and diabetes [24–26].

Leptin secreted by adipocytes suppresses food intake and stimulates energy expenditure and its levels are increased with adipogenesis and obesity [27, 28]. Moreover, intra- and extra-cellular levels of leptin are closely associated with adipocyte size, body fat mass and body weight, and it influenced by environmental factors or hormones such as insulin and DEX [29].

Adiponectin, also known as GBP28, apM1, Acrp30, or AdipoQ, is a 244-residue protein that is produced mainly by white adipose tissue (WAT) and plays an important role in maintaining energy homeostasis and insulin sensitivity [30]. It is induced by transcription factors such as PPARγ, C/EBPα and sterol regulatory element-binding protein 1c (SREBP-1c), which are involved in adipocyte differentiation [31]. Furthermore, adiponectin is induced by PPARγ agonists and regulates adipocyte differentiation through the PPAR response element [32, 33].

Resistin, an adipose tissue-specific secretory factor in rodents, is a cysteine-rich protein secreted from differentiated adipocytes and WAT [34, 35]. Previous studies have shown that circulating resistin levels are correlated with risk factor of MS such as type 2 diabetes mellitus, obesity, and rheumatoid arthritis [36, 37].

PAI-1, an inhibitor of fibrinolysis, is a serine protease inhibitor that generally inhibits tissue and urokinase-type plasminogen activators. It is upregulated with lipid accumulation and it has been reported that circulating PAI-1 is a risk factor of cardiovascular diseases, obesity, and type 2 diabetes mellitus [38–41].

In this study, the levels of adipokines such as leptin, adiponectin, resistin and PAI-1 markedly decreased following DHSMT treatment. These results suggest that DHSMT may function as a negative regulator of adipogenesis.

Adipocyte differentiation is a process that is regulated by the complex modulation of various transcription factors and extracellular proteins. The transcription factors PPARγ and members of the C/EBPs, which regulate adipogenesis and insulin sensitivity in adipocytes, are especially important [42, 43]. The activation of C/EBPβ and C/EBPδ, which are expressed earlier than both PPARγ and C/EBPα during early adipocyte differentiation, stimulates the expression of C/EBPα and PPARγ either singly or together [44]. Notably, PPARγ, one of the nuclear hormone receptors, has been shown to be necessary for adipogenesis. It is extensively stimulated in adipose tissue and stimulates the differentiation of preadipocytes to adipocytes [45]. It is also known to bind to the C/EBPα promoter region, which is regulated by C/EBPβ during adipocyte differentiation [46]. Moreover, transcriptional factors such as PPARγ and C/EBPα, regulate adipogenesis-specific genes, such as fatty acid synthase (FAS), fatty acid binding protein (FABP) and lipoprotein lipase (LPL) that is involved in maintaining adipogenesis [47]. Also, adipocyte differentiation that is modulated by adipogenic-specific transcription factors markedly increased the expression of termination markers such as adiponectin which can facilitate lipid accumulation during the late adipocyte differentiation stage [48]. Our results revealed that DHSMT considerably down-regulated the protein expression of PPARγ, C/EBPα and FABP4, which are essential for adipocyte differentiation and adipogenesis. Moreover, SB203580 as a positive control also significantly suppressed the protein expression of PPARγ, C/EBPα and FABP4. These results suggested that DHSMT and SB203580 significantly blocked adipocyte differentiation and lipid accumulation by suppressing adipogenic gene expression.

## Conclusions

In conclusion, results of our present study demonstrate that DHSMT has the inhibitory effects on adipogenesis in 3T3-L1 adipocytes by decreasing the accumulation of intracellular lipid and adipokines such as leptin, adiponectin, resistin and PAI-1 without any cytotoxicity. Furthermore, these anti-adipogenic effects of DHSMT may be mediated through the down-regulation of PPARγ, C/EBPα and FABP4 at the protein level. These findings suggest that DHSMT could be used therapeutically for the treatment and prevention of obesity or other MS-associated conditions that are related to BSS.

## Abbreviations

BSS: Blood stasis syndrome
C/EBPα: CCAAT/enhancer binding proteins alpha
CCK-8: Cell counting kit-8
DEX: Dexamethasone
DHSMT: Dohongsamul-Tang
DMEM: Dulbeco’s modified eagle’s medium
DMSO: Dimethyl sulfoxide
DPBS: Dulbeco’s phosphate-buffered saline
FABP4: Fatty acid binding protein 4
FAS: Fatty acid synthase
FBS: Fetal bovine serum
IBMX: 3-isobutyl-1-methylisobutylxanthine
LPL: Lipoprotein lipase
MDI: A mixture of 0.5 mM IBMX, 1 uM dexamethasone and 1 μg/ml insulin
MS: Metabolic syndrome
NBCS: Newborn calf serum
P&S: Penicillin-streptomycin
PAI-1: Plasminogen activator inhibitor-1
PPARγ: Proliferator-activated receptor gamma
SREBP-1c: Sterol regulatory element-binding protein 1c
TG: Trigliceride
TKM: Traditional Korean medicine
